# The first report on homozygous *INHA* inactivation in humans

**DOI:** 10.1530/EJE-22-0330

**Published:** 2022-04-29

**Authors:** Ilpo Huhtaniemi

**Affiliations:** 1 Department of Digestion, Metabolism and Reproduction, Institute of Reproductive and Developmental Biology, Hammersmith Campus, Imperial College London, London, UK; 2 Institute of Biomedicine, Research Centre for Integrative Physiology and Pharmacology, University of Turku, Turku, Finland

Inhibin A and B are heterodimeric proteins of inhibin alpha (INHA) and beta (INHBA or INHBB) subunits, and homodimers of two beta subunits form activins. They belong to the transforming growth factor beta superfamily of pleiotropic signaling molecules. While inhibins are mainly formed in gonads, activins are widely produced in extragonadal sites. Their classical action is to regulate positively (activin) or negatively (inhibin) pituitary secretion of follicle-stimulating hormone (FSH), but activins also have diverse non-endocrine effects, including cell proliferation, inflammation and carcinogenesis (1). Both proteins have multiple intragonadal paracrine and autocrine actions, related to hormone production and gametogenesis (1). The major mode of inhibin action is considered as inhibition of activin action at the activin receptor site (2).

Betaglycan, the receptor participating in inhibin’s inhibitory action on activin, is expressed in human testis in primary spermatocytes, round spermatids and in Leydig cells (LC), which are thus potential targets of intratesticular inhibin action (1). Activin receptors are expressed in multiple cell types of the human testis, including LC and Sertoli cells (SC), spermatogonia, spermatocytes and round spermatids (1). Overall, enhanced activin signaling inhibits LC function, and stimulates proliferation and inhibits differentiation of SC, and it reduces their capacity to maintain spermatogenesis (3). Inhibin functions within the testis mainly by counteracting the effects of activin.

A great deal about inhibin and activin functions has been learned from genetically modified mouse models, that is inhibin and activin knockouts (4, 5). *Inha* *^−/−^* mice develop infertility and gonadal sex cord stromal tumours, of SC origin in males, followed by a cachexia-like wasting syndrome (4). The phenotype suggested tumour-suppressor role for inhibin, but it appeared to be mostly due to the unopposed strong activin action in the absence of inhibin. This is proven by another recently produced knock-in mouse model, where a point mutation in *Inha* gene prevented the proteolytic activation of inhibin without perturbing normal activin action (6). While the female knock-in mice had severe subfertility with disturbed ovarian function, no phenotype was observed in the male counterparts. Hence, the strong phenotype of *Inha* knockout mice was apparently due to unopposed activin action, instead of inhibin elimination.

Very little is still known about the consequences of genetic inhibin and activin inactivation in humans, apart from some reports on heterozygous mutations associated with premature ovarian failure and gonadal tumours (7, 8). The effect of complete inhibin inactivation in the human has so far remained unknown. Therefore, the interesting report of Arslan Ates *et al.* (9) on two consanguineous Turkish brothers (parents first-degree cousins) with homozygous *INHA* mutation provides totally new information about the functions of inhibin and activin in humans. The brothers presented with hypospadias, gynecomastia, primary hypergonadotrophic testicular failure, large testis size and azoospermia. Hormone measurements demonstrated elevated luteinizing hormone (LH) and FSH levels, low testosterone, highly elevated anti-Müllerian hormone (AMH) and very low to nondetectable inhibin B. Both men were found to carry a homozygous 2-bp deletion (c.208_209delAG, R70Gfs*3) in the *INHA* gene, resulting in frameshift of the message and truncation of the INHA protein. Although functional studies on the mutant gene were not carried out, it is likely it resulted in complete loss of inhibin production. Unfortunately, due to logistic problems, activin levels could not be measured. Circumstantial evidence for increased activin action at the pituitary levels was provided by the strongly elevated FSH levels. Testis biopsy was not carried out.

The phenotype of the men can be explained by current knowledge of inhibin and activin actions, mainly for animal models. Strong activin action has been shown to stimulate SC proliferation but inhibit their maturation (10). This may explain the failure of spermatogenesis, critically dependent on support by mature SC function. Further evidence for SC maturation defect is provided by the strongly elevated levels of AMH, which is a hormone produced only by immature SC (11). The high FSH levels were due to missing inhibin and unopposed activin action at the pituitary level, and together with activin they were accountable for the large size of testes. Finally, elevated LH was a consequence of impaired LC function under the inhibition of activin. Inhibin of SC origin normally stimulates LC testosterone formation by blocking the inhibitory action of activin on 17-hydoxylase/C17-20 lyase (11), which can explain the suppressed testosterone levels in the inhibin-deficient brothers. Moreover, AMH has direct inhibitory action on LC steroidogenesis (12). Impaired androgen production also explained hypospadias of the brothers, and their gynaecomastia can be explained by elevated estrogen/androgen ratio.

The men, unlike *Inha* knockout mice, had no signs of testicular tumours or cachexia. It will be warranted to keep these conditions in mind upon follow-up of the patients because we do not know whether they represent a genuine species difference or may take longer to appear in humans. Long-term follow-up of the brothers will undoubtedly reveal us more about the effects of missing inhibin action. The histology of large testes devoid of complete spermatogenesis would be of particular interest, if testicular sperm extraction were to be used for infertility treatment. Human chorionic gonadotrophin treatment, in order to increase intratesticular testosterone levels, is unlikely to cure azoospermia, because of the activin-induced maturation arrest of SC. Attempts to suppress activin action would probably be more promising. Maybe the novel inhibitors of the activin receptor signaling pathway could offer a solution for reversing the azoospermia (13).

## Declaration of interest

The authors declare that there is no conflict of interest that could be perceived as prejudicing the impartiality of the research reported.

## Funding

This work did not receive any specific grant from any funding agency in the public, commercial, or not-for-profit sector.
